# Uterine Leiomyomas with Specific Histology Features of Two Fumarate Hydratase/Succinate Dehydrogenase-Deficient Tumors: A Double Case Report

**DOI:** 10.3390/medicina60050825

**Published:** 2024-05-17

**Authors:** Ljubiša Jovanović, Svetlana Milenković, Luka Andrić, Radomir Stefanović, Branislav Milošević, Jelena Micić, Igor Pilić, Aleksandra Beleslin, Olga Mihaljević, Milan Dokić

**Affiliations:** 1 Department of Pathology and Medical Cytology, University Clinical Center of Serbia, Dr. Koste Todorovića 26, 11000 Belgrade, Serbia; cecana63@gmail.com (S.M.); r_stefanovic@hotmail.com (R.S.); 2 Clinic for Gynecology and Obstetrics, University Clinical Center of Serbia, 11000 Belgrade, Serbia; lukaandric9@gmail.com (L.A.); drbanemilosevic@gmail.com (B.M.); jdmicic@yahoo.com (J.M.); pilic.igor@yahoo.com (I.P.); aleksandrabeleslin@gmail.com (A.B.); oollga94@gmail.com (O.M.); milanddokic@gmail.com (M.D.); 3 Medical Faculty, University of Belgrade, 11000 Belgrade, Serbia

**Keywords:** leiomyoma, fumarate, succinate deficiency

## Abstract

*Background and Objectives*: Mutations in succinate dehydrogenase (SDH) and fumarate hydratase (FH) give rise to various familial cancer syndromes, with these alterations being characteristic of certain types of histomorphologically specific leiomyomas that hold significant predictive value. *Materials and Methods*: This study presents two cases of uterine leiomyomas exhibiting rare histomorphological and genetic characteristics, which are crucial for prognosis and further treatment. *Results*: Distinct histopathological features such as marked nuclear atypia, intracellular eosinophilic globules, and abnormal intratumoral vessels raise suspicion for specific leiomyoma subtypes, which carry predictive significance for additional hereditary cancer syndromes. Immunohistochemical analysis confirmed FH/SDH deficiency in both patients, who underwent careful follow-up. *Conclusions*: This study describes two cases involving unusual leiomyomas, the histopathological characteristics of which may easily go unrecognized. These features hold predictive significance because their specific mutations point to additional hereditary cancer syndromes, highlighting the need for further examinations.

## 1. Introduction

Leiomyomas, which are benign smooth muscle tumors prevalent within the female genital tract, are a common occurrence, affecting approximately 70% of women by the age of 50, as documented by various studies [1,2,3]. There are many different histological types with various specific patterns. In recent years, new histological types of leiomyomas have been discovered, expanding our understanding of their morphological diversity and potentially augmenting their predictive capabilities [1].

Different hereditary cancer syndromes are rooted in mutations in metabolic pathways. They typically involve multiple tumors in various locations, exhibiting diverse biological behaviors. Cancer cell metabolic functions are crucial for tumor progression and clinical presentation. Dysfunctional metabolic processes often contribute to early tumor development and uncommon clinical features [1,2].

Recent studies have indicated that specific histological types of leiomyomas are associated with aggressive forms of renal cell carcinomas [1,2]. These affect up to 300 families annually. In these syndromes, smooth muscle tumors are usually benign, while renal tumors are highly progressive carcinoma types [2,3].

Mutations in succinate dehydrogenase (SDH) and fumarate hydratase (FH) cause various familial cancer syndromes. These enzymes play crucial roles in the mitochondrial respiratory chain and in regulating the Krebs cycle. Mutations in FH result in decreased enzyme activity and the accumulation of fumarate, leading to mitochondrial inactivity. SDH consists of four subunits, with the B unit (SDHB) being the most studied and the most capable of representing the metabolic mechanism [1].

FH functions as a vital enzyme in the Krebs cycle, facilitating the conversion of fumarate to malate. This enzyme exhibits a high degree of conservation across various organisms, encompassing both prokaryotes and eukaryotes, including in humans. Apart from its role in the Krebs cycle, FH also plays a significant role in the DNA damage response pathway. In humans, FH is encoded by the FH gene, which is situated on the q-arm of chromosome 1 (chr1q43). Mutations that lead to FH inactivation can give rise to two distinct disorders: fumarate hydratase deficiency, an autosomal recessive disorder of metabolism, and hereditary leiomyomatosis and renal cell carcinoma (HLRCC), an autosomal dominant cancer syndrome with incomplete penetrance. These two disorders exhibit contrasting clinical presentations. Fumarate hydratase deficiency typically manifests during the neonatal to early infantile stages, and is characterized by symptoms such as encephalopathy, failure to thrive, and various neurological abnormalities. In contrast, HLRCC often becomes apparent during adolescence or adulthood, with the likelihood of disease manifestation increasing with age. HLRCC is typified by the development of multiple tumor types, including skin leiomyomas, uterine fibroids, and kidney tumors resembling type 2 papillary renal cell carcinoma. Additionally, bladder cancer and Leydig cell tumors of the testis have been documented in HLRCC patients. Skin leiomyomas and uterine fibroids associated with HLRCC are benign tumors that are prone to recurrence, posing significant challenges and causing discomfort for affected individuals. Moreover, HLRCC-associated renal cancer represents an exceptionally aggressive form of kidney cancer, necessitating urgent attention and effective treatment strategies [1,2,3,4].

In this study, we emphasize the significance of well-recognized fumarate/succinate-deficient leiomyoma subtypes due to their substantial prognostic and diagnostic impact.

## 2. Materials and Methods

This study presents two cases of uterine leiomyomas with rare histomorphological and genetic characteristics, which are significant for the prognosis and further treatment of such patients. The patients were 25 and 28 years old, respectively, and both were treated at a gynecological clinic after an incidental finding of uterine leiomyoma.

The specimens underwent thorough review during macroscopic examination. The tissues were fixed in 10% buffered formalin and embedded in paraffin. An evaluation of the histopathological patterns was conducted on 4 µm thick, fully hematoxylin and eosin (H&E)-stained sections, using a Leica 2000 microscope, following the current World Health Organization (WHO, Geneva, Switzerland) classification guidelines.

From each case, the most representative samples were selected for immunohistochemical analysis. Immunohistochemical staining for both SDHB and FH antibodies was performed on the Autostainer Link 48, Agilent, Denmark, according to routine standards and the manufacturer’s recommendations. For SDHB, we used a mouse monoclonal antibody (clone ab14714, dilution 1:100, Abcam, Boston, MA, USA). FH immunohistochemical analysis was conducted using a mouse monoclonal antibody (clone J-13, dilution 1:50, Santa Cruz, Dallas, TX, USA). Heat-induced epitope retrieval was carried out using the Universal DAB detection system with Ventana Cell Conditioning 1 (pH 8.4) at 97 °C for 52 and 92 min, respectively, for the SDHB antibody; and using CC1 at 95 °C for 36 min followed by antibody incubation at 37 °C for 32 min for the FH antibody. The ultraView visualization system was utilized for immunohistochemical analysis [5,6]. Immunohistochemical assessment was conducted by two certified pathologists. A granular cytoplasmic staining pattern (mitochondrial staining) was considered valuable. Reactions that were positive for certain mutations were characterized by the presence of SDHB positivity and the absence of FH immunohistochemical reaction. Endothelial cells served as an internal positive control [5,6].

## 3. Results

### 3.1. Clinical Features

Our patients presented with similar clinical and imaging features. The initial symptom for both patients was irregular menstrual bleeding over the past few years. Following routine gynecological examinations and ultrasound imaging, large uterine tumor masses suspected to be leiomyomas were diagnosed in both patients. The first patient had a remarkable tumor in the uterine wall, measuring 80 mm in diameter with regular borders. The tumor deformed the uterine walls, compressing the bladder and surrounding anatomical structures. In the second patient, the tumor was smaller, approximately 40 mm in size, but when ultrasounds were performed, rapid growth was observed over a three-month period, suggesting a need for operative treatment. Examination of the endometrium, ovaries, tubes, and cervical tissue revealed no suspicious tumor features in either patient.

In the family history of our patients, no associated conditions or diseases were recognized regarding specific autosomal recessive (AR) inheritance patterns for these particular types of leiomyomas and certain mutations. However, the first patient reported receiving a diagnosis of breast carcinoma a couple of years ago.

**Figure 1:**
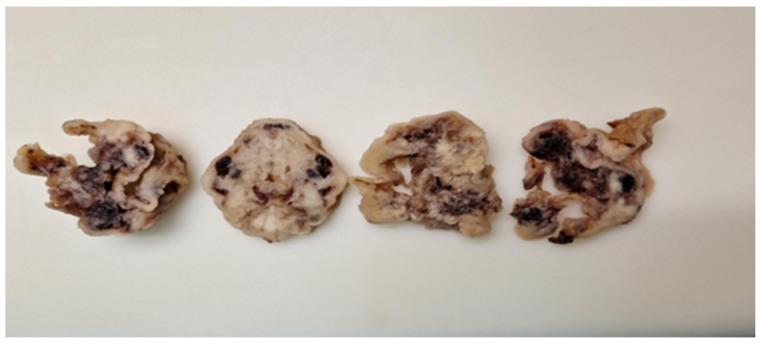
Extirpated leiomyomas displaying no specific macroscopic features. Multifocal hemorrhagic spots were observed within the solid tissue.

Both patients underwent myomectomy via laparotomy. Solitary leiomyomas were excised (Figure 1). There were no clinical or imaging indications of surrounding infiltration. The leiomyomas were well demarcated with benign clinical and radiological characteristics. However, the second patient experienced more extensive intraoperative bleeding than is usually expected. The postoperative course for both surgeries was uneventful, and the patients were discharged on the fifth postoperative day.

### 3.2. Histopathological Characteristics

The results of hematoxylin and eosin (H&E) staining unveiled several remarkable histomorphological features in these leiomyoma subtypes, which were strongly suggestive of their classification. These features encompassed nuclear pleomorphism, characterized by multinucleation or the presence of large, hyperchromatic, or irregular nuclear forms, as well as prominent eosinophilic nucleoli surrounded by a perinuclear halo, eosinophilic globules, hydropic degeneration, and hyalinization. Cellularity, a significant parameter, was assessed and categorized as low, moderate, or high. The mitotic activity was evaluated by observing 10 high-power fields (HPFs) in a hotspot exhibiting the most increased cellularity.

**Figure 2:**
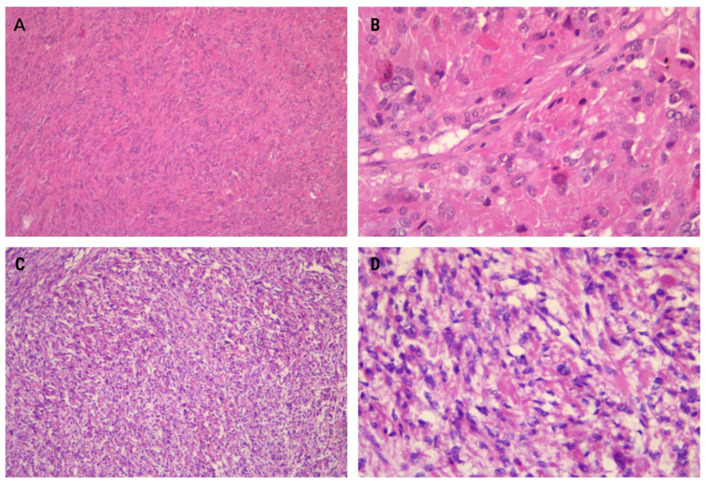
Specific histomorphological features of the presented leiomyomas include a spindle-like arrangement of tumor cells, which is a common distinctive feature (**A**) ×100. At higher magnification, eosinophilic cytoplasmic globules are also visible (**B**) ×400. The leiomyomas exhibit hypercellular features with vague nuclear palisading (**C**) ×100. Prominent nuclear pleomorphism and the presence of multinucleated cells are notable features. Additionally, some nuclei exhibit pseudoinclusions and eosinophilic cytoplasmic globules (**D**) ×400.

In our first case, moderate cellularity was observed, accompanied by distinctive spindle-like arrangements of tumor cells embedded within a diffuse collagenous matrix. The tumor exhibited elongated cells arranged in long sweeping to poorly formed fascicles (Figure 2A). Mildly enlarged eosinophilic nucleoli, eosinophilic cytoplasmic inclusions, and staghorn vessels were additional notable features. Mitotic activity was lower compared to the second case (1/10HPF), and remarkable nuclear atypia was absent (Figure 2B).

The second case demonstrated higher cellularity than typical uterine leiomyomas (Figure 2C). Mitotic activity was slightly elevated compared to the first case (2/10HPF). Multinucleation, often focal with wreath-like nuclear arrangements and nuclear pseudoinclusions, was a common finding. Another notable observation was the significant microvessel density (Figure 2D).

### 3.3. Immunohistochemistry Features

**Figure 3:**
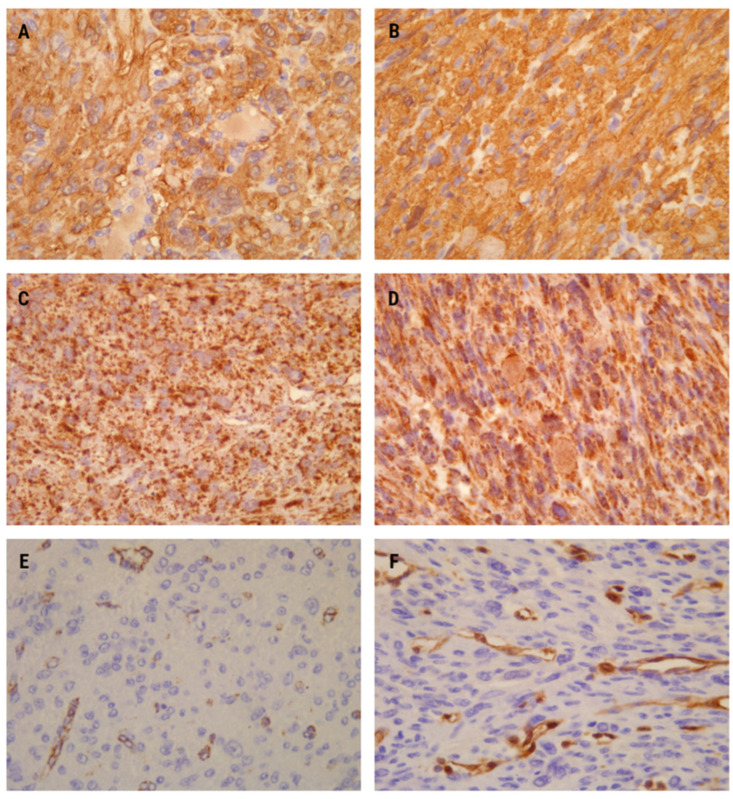
Immunohistochemistry analysis showed diffuse positivity for smooth muscle actin (SMA) in both cases (**A**,**B**) ×400. SDHB immunopositivity exhibited strong and diffuse staining (dot-like and granular) in the cytoplasm and mitochondria in both patients (**C**,**D**) ×400. Fumarate hydratase (FH) immunonegativity was observed in the first (**E**) ×400 and second cases (**F**) ×400 as a complete absence of staining in tumor cells. Immunopositivity for FH in vessels (endothelial cells and intima) served as an internal positive control.

The performed immunohistochemical analysis showed very similar expression patterns in both presented cases. Markers specific for smooth muscle differentiation confirmed the histogenesis of both tumors. Both leiomyomas revealed positive staining for smooth muscle actin (SMA), confirming their smooth muscle origin (Figure 3A,B). Additionally, heavy caldesmon and desmin were also found to be positive. Progesterone receptor-positivity was a typical feature observed in both cases, while CD10 was notably negative. The tumor proliferation was assessed to be low. When compared to non-FH-deficient conventional leiomyomas, the immunostaining patterns in both cases remained similar.

The first leiomyoma exhibited a specific dot-like immunostaining pattern for the SDHB antibody. This intracytoplasmic staining appeared granular, involving more than 90% of histomorphologically suspicious smooth muscle cells (Figure 3C). Conversely, FH expression was lost in the majority of tumor cells (Figure 3E).

In the second leiomyoma, SDHB immunohistochemical expression was retained, appearing slightly stronger than in the first one. The staining was also cytoplasmic, representing mitochondria with certain genetic alteration. However, the nuclei remained unstained (Figure 3D). Conversely, no FH positivity was observed in the leiomyoma cells. Similar to the first case, positive FH staining was present only in vascular endothelial cells, with vascular smooth muscle cells serving as internal positive controls for the immunohistochemical analysis.

Based on the comprehensive assessment integrating histomorphological and immunohistochemistry findings, we rendered a diagnosis of uterine leiomyomas with strongly suspected FH deficiency. Following surgery, the general condition of both patients was significantly improved without any signs of their previous symptoms. They were referred to a gynecologist for follow-up and consideration of further genetic testing for specific SDHB/FH mutations necessary for a definitive diagnosis.

During a one-year follow-up period, ultrasonography revealed no signs of leiomyoma recurrence. However, it is important to note that we lacked data regarding any positive genetic results for certain mutation types, underscoring the potential importance of genetic testing in confirming the diagnosis and guiding future management decisions.

## 4. Discussion

Uterine leiomyomas exhibit various histological features. Leiomyomas with FH/SDH deficiency manifest specific histological characteristics that should prompt further immunohistochemical analysis and confirmation of the diagnosis [2].

In this study, we describe two cases with similar clinical, histopathological, and immunohistochemistry characteristics. Both patients were young. The leiomyomas were solitary, with larger diameters and nonspecific clinical features. FH (−) and SDHB (+) expression in both leiomyomas pointed to FH deficiency.

Immunohistochemistry expressions of FH and SDHB antibodies are widely used to detect FH deficiency. Loss of FH expression indicates the presence of mutations in tumor cells, with SDHB applied as an additional marker to confirm the results of FH loss [7]. Our tumor cells exhibited positive granular, cytoplasmic reactions for SDHB and loss of FH expression, with FH+ endothelial cells serving as a positive internal control.

Nearly all women with HLRCC have uterine leiomyomas, typically multiple and large in size, presenting at early ages. The median age of patients presenting with FH-deficient leiomyomas is approximately ten years younger than that of patients presenting with sporadic ones [2,8]. In our study, both leiomyomas were solitary but still occurred in patients at a young age and were quite large.

Studies have reported a strong association between leiomyomas with bizarre nuclei, notable cytonuclear atypia, and FH deficiency [2,5]. FH deficiency can sporadically occur in some leiomyosarcomas and is rare in conventional leiomyomas, emphasizing the need for careful distinction between leiomyoma with bizarre nuclei, leiomyosarcoma, and smooth muscle neoplasms of uncertain malignant potential (STUMP). Both of our cases support the correlation between bizarre histological leiomyoma characteristics and FH/SDH deficiency [5].

In addition to nuclear features, specific histological features have been described for these leiomyomas, including dilated staghorn vessels, perinuclear halos, and eosinophilic cytoplasmic inclusions, which are quite specific to leiomyoma with FH deficiency [2,9], similar to that observed in our cases.

Early detection of FH-deficient tumors is crucial due to their hereditary association. Leiomyomas with FH/SDH deficiency could serve as the initial manifestation of hereditary cancer syndromes. Careful management and follow-up of these patients may be necessary. Patients with FH mutations should undergo annual magnetic resonance imaging screenings. Detected renal tumors require treatment in the form of radical surgical procedures. The significance of this study lies in raising awareness of this histological type of leiomyomas and prompting initial suspicion for possible cancer development in the future for patients who receive this diagnosis [10,11].

Recent studies have described several hereditary cancer syndromes, with specific leiomyomas being among the reported tumors [2,4,10]. The World Health Organization (WHO) in 2016 classified a new histological entity as Hereditary Leiomyomatosis and Renal Cell Cancer (HLRCC), where papillary renal cell carcinoma type II was the histology type most commonly associated with uterine leiomyomas. Considering SDHB and FH mutations, recent renal cell tumor classifications have incorporated a few different entities according to molecular profiles specific to certain histopathological features. Succinate dehydrogenase (SDH)-deficient renal cell carcinoma (RCC) and fumarate hydratase-deficient RCC (FH-deficient RCC) were implicated [10]. The same types of genetic alterations were detected in specific leiomyoma types described in this study. Patients with such leiomyomas have a five-to-six-times increased risk of developing aggressive forms of renal cell carcinomas [2].

Tumor tissues consistently exhibit a loss of heterozygosity at the FH locus, resulting in a complete loss of FH enzymatic function. Extensive research over the past two decades has shed light on the molecular pathways influenced by FH inactivation, paving the way for targeted treatment approaches aimed at FH-deficient tumor cells. These therapeutic avenues encompass techniques such as inducing ferroptosis, promoting oxidative stress, and altering metabolic processes. As our understanding of HLRCC deepens, we are optimistic that these treatment modalities will undergo further refinement, potentially offering strategies for preventing the onset of HLRCC. With continued advancements in HLRCC biology, the translation of experimental treatments into clinical applications in the near future appears increasingly feasible [4,12,13].

Biallelic FH inactivation induces significant changes in the transcriptome and proteome. Since biallelic FH inactivation is exclusive to cancer cells in HLRCC patients, the biological alterations driven by FH inactivation present unique opportunities for targeted therapy. Prior to the molecular characterization of HLRCC tumors, targeted therapies for this aggressive tumor type primarily focused on the metabolic changes caused by FH inactivation. Specifically, FH inactivation increases the cellular reliance on glycolysis for ATP production. Concurrently, this metabolic shift leads to an increase in the production of reactive oxygen species. Consequently, HLRCC cells exhibit heightened sensitivity to the proteasome inhibitor bortezomib, which elevates cellular ROS levels and prompts apoptotic cell death. However, such therapies are poorly tolerated and have not yielded observable tumor regression [4,14].

The functional resemblance between SDH and FH enzymes, coupled with the analogous epigenetic alterations triggered by the accumulation of associated oncometabolites, suggests a potential genomic and metabolic similarity between SDH and FH-altered RCC. Recent investigations into FH-altered RCC have described a notably low tumor mutation burden, distinctive tumor microenvironments characterized by the infiltration of CD8+ T-cells with PD-L1 expression within tumors, and a profoundly depleted and mutated mitochondrial genome. In contrast, previous studies on SDH-altered RCC tumors have primarily focused on the clinical and pathological features of the disease and genetic analyses of specific SDH mutant alleles, without providing a comprehensive overview of the genomic landscape. Consequently, there has been limited exploration into the comprehensive molecular and metabolomic profiling of SDH mutated tumors [15,16].

The methods used to screen and identify inherited tumor syndromes among patients with uterine leiomyomas lack precision. The involvement of gynecologists and pathologists is pivotal in promptly diagnosing the syndrome. Detecting HLRCC syndrome at an early stage is crucial, since severely symptomatic uterine leiomyomas tend to occur at a significantly younger age compared to non-syndromic leiomyomas. While certain morphological features of FH-deficient uterine leiomyomas have been outlined, these features are not exclusive to syndromic FH mutations but also occur with HLRCC-unrelated somatic FH mutations. Additionally, the reliability and diagnostic accuracy of morphology in identifying FH-mutated leiomyomas are not yet optimal in terms of sensitivity and specificity [17,18].

Immunohistochemistry for FH, which typically exhibits complete loss of staining in FH-deficient tissue, is not recommended for ruling out HLRCC. This is because retained FH staining has been observed in uterine leiomyomas with an FH germline mutation. FH-deficient cells accumulate fumarate protein at high levels, and it spontaneously reacts with cysteine sulfhydryl groups to form S-(2-succinyl) cysteine (2SC). Detection of succinated proteins using the 2SC antibody serves as a biomarker for FH-deficient uterine leiomyomas. However, the commercial availability of the 2SC antibody is recent, and due to the rarity of HLRCC syndrome, most pathology laboratories may not have access to this antibody and staining. As a result, a definitive diagnosis of FH-deficient leiomyomas and HLRCC syndrome is typically established in specialized centers, while initial leiomyoma diagnosis and suspicion of FH-deficient leiomyomas occur in hospitals that may lack the resources to conduct a direct or indirect analysis of mutational status [12,18,19].

The limitations of this study include the absence of additional markers and correlation methods to strengthen the confirmation of our results. Unpredictable missense mutations in the FH gene could lead to the production of aberrant proteins and sometimes result in unrepresentative immunoexpression for these markers [10]. Nevertheless, studies have confirmed that the majority of leiomyomas with such bizarre features exhibit reliable specific FH immunoexpression, indicating its deficiency [5,8].

Careful selection of histologically specific leiomyomas is essential for effectively screening these mutated enzymes. Molecular genetic testing represents a subsequent procedure to confirm the presence of the described entities [8,19,20].

## 5. Conclusions

Uterine leiomyomas with such characteristics are rare and can easily go unrecognized, necessitating heightened awareness due to their prognostic significance. This type of leiomyoma warrants further diagnostic and therapeutic procedures for patients to prevent the development of aggressive tumor forms in other locations. Genetic testing and close clinical follow-ups are highly recommended for patients with such leiomyomas.

## Data Availability

Data are contained within the article.

## References

[1] Vilos G.A., Allaire C., Laberge P.Y., Leyland N., SPECIAL CONTRIBUTORS (2015). The management of uterine leiomyomas. J. Obstet. Gynaecol. Can..

[2] Saxena N., Maio N., Crooks D.R., Ricketts C.J., Yang Y., Wei M.H., Fan T.W., Lane A.N., Sourbier C., Singh A. (2016). SDHB-Deficient Cancers: The Role of Mutations That Impair Iron Sulfur Cluster Delivery. J. Natl. Cancer Inst..

[3] Huang Y., Zhou Y., Chen X., Fang Q., Cai H., Xie M., Xing Y. (2021). Uterine leiomyoma with fumarate hydratase deficiency: A case report. Medicine.

[4] Ooi A. (2020). Advances in hereditary leiomyomatosis and renal cell carcinoma (HLRCC) research. Semin. Cancer Biol..

[5] Siegler L., Erber R., Burghaus S., Brodkorb T., Wachter D., Wilkinson N., Bolton J., Stringfellow H., Haller F., Beckmann M.W. (2018). Fumarate hydratase (FH) deficiency in uterine leiomyomas: Recognition by histological features versus blind immunoscreening. Virchows Arch..

[6] Papathomas T.G., Oudijk L., Persu A., Gill A.J., van Nederveen F., Tischler A.S., Tissier F., Volante M., Matias-Guiu X., Smid M. (2015). SDHB/SDHA immunohistochemistry in pheochromocytomas and paragangliomas: A multicenter interobserver variation analysis using virtual microscopy: A Multinational Study of the European Network for the Study of Adrenal Tumors (ENS@T). Mod. Pathol..

[7] Lee H., Shafiezadeh S., Singh R. (2020). Fumarase-deficient uterine leiomyoma: A case of a rare entity and surgical innovation. J. Surg. Case Rep..

[8] Bennett J.A., Weigelt B., Chiang S., Selenica P., Chen Y.B., Bialik A., Bi R., Schultheis A.M., Lim R.S., Ng C.K.Y. (2017). Leiomyoma with bizarre nuclei: A morphological, immunohistochemical and molecular analysis of 31 cases. Mod. Pathol..

[9] Gregová M., Hojný J., Němejcová K., Bártů M., Mára M., Boudová B., Laco J., Krbal L., Tichá I., Dundr P. (2020). Leiomyoma with Bizarre Nuclei: A Study of 108 Cases Focusing on Clinicopathological Features, Morphology, and Fumarate Hydratase Alterations. Pathol. Oncol. Res..

[10] Liu C., Dillon J., Beavis A.L., Liu Y., Lombardo K., Fader A.N., Hung C.F., Wu T.C., Vang R., Garcia J.E. (2020). Prevalence of somatic and germline mutations of Fumarate hydratase in uterine leiomyomas from young patients. Histopathology.

[11] Zhang X., Wang C., Shen D. (2024). The use of Clinicopathological, immunohistochemistry and molecular detection in the diagnosis of fumarate hydratase-deficient uterine leiomyomas. Pathol. Res. Pract..

[12] Shi W., Liu Y., Aisagbonhi O., Roma A.A., Hasteh F., Zare S.Y., Fadare O. (2024). Fumarate Hydratase-Deficient Leiomyoma of the Uterine Corpus: Comparative Morphologic Analysis of Protein-Deficient Tumors with and without Pathogenic Germline Fumarate Hydratase Gene Mutations. Int. J. Surg. Pathol..

[13] Kopp R.P., Stratton K.L., Glogowski E., Schrader K.A., Rau-Murthy R., Russo P., Coleman J.A., Offit K. (2017). Utility of prospective pathologic evaluation to inform clinical genetic testing for hereditary leiomyomatosis and renal cell carcinoma. Cancer.

[14] Park I., Shim Y.S., Go H., Hong B.S., Lee J.L. (2019). Long-term response of metastatic hereditary leiomyomatosis and renal cell carcinoma syndrome associated renal cell carcinoma to bevacizumab plus erlotinib after temsirolimus and axitinib treatment failures. BMC Urol..

[15] Yoo A., Tang C., Zucker M., Fitzgerald K., DiNatale R.G., Rappold P.M., Weiss K., Freeman B., Lee C.H., Schultz N. (2022). Genomic and Metabolic Hallmarks of SDH- and FH-deficient Renal Cell Carcinomas. Eur. Urol. Focus.

[16] Gleeson J.P., Nikolovski I., Dinatale R., Zucker M., Knezevic A., Patil S., Ged Y., Kotecha R.R., Shapnik N., Murray S. (2021). Comprehensive Molecular Characterization and Response to Therapy in Fumarate Hydratase-Deficient Renal Cell Carcinoma. Clin. Cancer Res..

[17] Trpkov K., Hes O., Agaimy A., Bonert M., Martinek P., Magi-Galluzzi C., Kristiansen G., Lüders C., Nesi G., Compérat E. (2016). Fumarate Hydratase-deficient Renal Cell Carcinoma Is Strongly Correlated with Fumarate Hydratase Mutation and Hereditary Leiomyomatosis and Renal Cell Carcinoma Syndrome. Am. J. Surg. Pathol..

[18] Uimari O., Ahtikoski A., Kämpjärvi K., Butzow R., Järvelä I.Y., Ryynänen M., Aaltonen L.A., Vahteristo P., Kuismin O. (2021). Uterine leiomyomas in hereditary leiomyomatosis and renal cell cancer (HLRCC) syndrome can be identified through distinct clinical characteristics and typical morphology. Acta Obstet. Gynecol. Scand..

[19] Miettinen M., Felisiak-Golabek A., Wasag B., Chmara M., Wang Z., Butzow R., Lasota J. (2016). Fumarase-deficient Uterine Leiomyomas: An Immunohistochemical, Molecular Genetic, and Clinicopathologic Study of 86 Cases. Am. J. Surg. Pathol..

[20] Chan M.M.Y., Barnicoat A., Mumtaz F., Aitchison M., Side L., Brittain H., Bates A.W.H., Gale D.P. (2017). Cascade Fumarate Hydratase mutation screening allows early detection of kidney tumour: A case report. BMC Med. Genet..

